# Variability in cardiac electrophysiology: Using experimentally-calibrated populations of models to move beyond the single virtual physiological human paradigm

**DOI:** 10.1016/j.pbiomolbio.2015.12.002

**Published:** 2016-01

**Authors:** Anna Muszkiewicz, Oliver J. Britton, Philip Gemmell, Elisa Passini, Carlos Sánchez, Xin Zhou, Annamaria Carusi, T. Alexander Quinn, Kevin Burrage, Alfonso Bueno-Orovio, Blanca Rodriguez

**Affiliations:** a Department of Computer Science, University of Oxford, Parks Road, Oxford OX1 3QD, United Kingdom; b Clyde Biosciences Ltd, West Medical Building, University of Glasgow, Glasgow G12 8QQ, United Kingdom; c Center for Computational Medicine in Cardiology (CCMC), Institute of Computational Science, Università della Svizzera italiana, Lugano, Switzerland; d Medical Humanities, University of Sheffield, United Kingdom; e Department of Physiology and Biophysics, Dalhousie University, Halifax, Nova Scotia, Canada; f Mathematical Sciences, Queensland University of Technology, Queensland 4072, Australia; g ACEMS, ARC Centre of Excellence for Mathematical and Statistical Frontiers, Queensland University of Technology, Queensland 4072, Australia

**Keywords:** Physiological variability, Cardiac electrophysiology, Populations of models, Action potential, *In silico* high-throughput screening, Arrhythmias

## Abstract

Physiological variability manifests itself via differences in physiological function between individuals of the same species, and has crucial implications in disease progression and treatment. Despite its importance, physiological variability has traditionally been ignored in experimental and computational investigations due to averaging over samples from multiple individuals. Recently, modelling frameworks have been devised for studying mechanisms underlying physiological variability in cardiac electrophysiology and pro-arrhythmic risk under a variety of conditions and for several animal species as well as human. One such methodology exploits populations of cardiac cell models constrained with experimental data, or experimentally-calibrated populations of models. In this review, we outline the considerations behind constructing an experimentally-calibrated population of models and review the studies that have employed this approach to investigate variability in cardiac electrophysiology in physiological and pathological conditions, as well as under drug action. We also describe the methodology and compare it with alternative approaches for studying variability in cardiac electrophysiology, including cell-specific modelling approaches, sensitivity-analysis based methods, and populations-of-models frameworks that do not consider the experimental calibration step. We conclude with an outlook for the future, predicting the potential of new methodologies for patient-specific modelling extending beyond the single virtual physiological human paradigm.

## 1 Introduction

Physiological variability manifests itself through differences in physiological function between individuals of the same species (Britton et al., 2013, Marder and Taylor, 2011, Sarkar et al., 2012). In cardiac electrophysiology, there are significant inter-subject and intra-subject differences in the electrical activity of cardiac tissue from the same region of the heart (Feng et al., 1998, Walmsley et al., 2015). At the level of isolated cardiac cells (cardiomyocytes), variability becomes apparent via differences in the morphology and duration of their electrical signal – the action potential (AP).

One cause of variability is the biophysical processes responsible for the flow of ionic currents across the cellular membrane. Multiple proteins regulate the sarcolemmal flow of ionic species vital for electrophysiological function, including sodium, calcium, and potassium ions, and an alteration in the balance of these ionic currents would give rise to differences in the AP. Crucially, these currents are affected by processes such as protein expression (Schulz et al., 2006), cell environment (Severi et al., 2009, Vincenti et al., 2014), and circadian rhythms (Jeyaraj et al., 2012, Ko et al., 2009). Therefore, even for a specific cell, the balance of ionic currents will change in time or under drug action and following the onset of disease.

Physiological variability has significant implications for treating and managing heart diseases. For instance, drugs that are designed to have anti-arrhythmic properties in a diseased tissue, at certain heart rates, and with a particular acid-base balance, can become pro-arrhythmic at different heart rates or in less diseased tissue (Savelieva and Camm, 2008). Likewise, susceptibility to pathological conditions such as arrhythmias can also differ from individual to individual or depending on the condition of the patient (Severi et al., 2009, Vincenti et al., 2014). By studying variability, we can explore and improve our understanding of the mechanisms that lead to differences in outcomes when different individuals have the same condition or are given the same treatment.

Physiological variability is difficult to investigate with experimental methods alone (Carusi et al., 2012, Sarkar et al., 2012) due to the need to average data to control experimental error. Recently, a body of research (Britton et al., 2013, Groenendaal et al., 2015, Sarkar et al., 2012) has shown the power of computer models for investigations into the sources and modulators of biological variability. Specifically, populations of models – also referred to as ensembles of models – have proven useful in investigations of cardiac electrophysiological variability as reviewed by (Sarkar et al., 2012). Recent studies have furthered the methodology by explicitly incorporating experimental data into the construction of populations of models, thus yielding *experimentally-calibrated populations of models* (Britton et al., 2014, Britton et al., 2013, Muszkiewicz et al., 2014, Passini et al., 2015, Sánchez et al., 2014, Zhou et al., 2013).

The main aim of this paper is to review recent insights into variability in cardiac electrophysiology obtained through experimentally-calibrated populations of models in a variety of cell types and species. We discuss the ability of the experimentally-calibrated population-of-models methodology to provide new insights into sources and implications of variability in cardiac electrophysiology in physiological and pathological conditions, and following pharmacological interventions. The paper presents a description of the methodology and its comparison with alternative approaches for studying variability in cardiac electrophysiology, including cell-specific modelling (Davies et al., 2012, Groenendaal et al., 2015, Syed et al., 2005), sensitivity-analysis-based methods (Pueyo et al., 2010, Romero et al., 2009, Sobie and Sarkar, 2011, Sobie, 2009), and population-of-models methods without experimental calibration (Cummins et al., 2014, Devenyi and Sobie, 2015, Sarkar et al., 2012, Walmsley et al., 2013, Yang and Clancy, 2012). We conclude with an outlook for the future, predicting the potential of new methodologies for patient-specific modelling beyond the single virtual physiological human paradigm. This paper is part of the special issue on Recent Developments in Biophysics & Molecular Biology of Heart Rhythm.

## 2 Description of the experimentally-calibrated population-of-models methodology

**Fig. 1:**
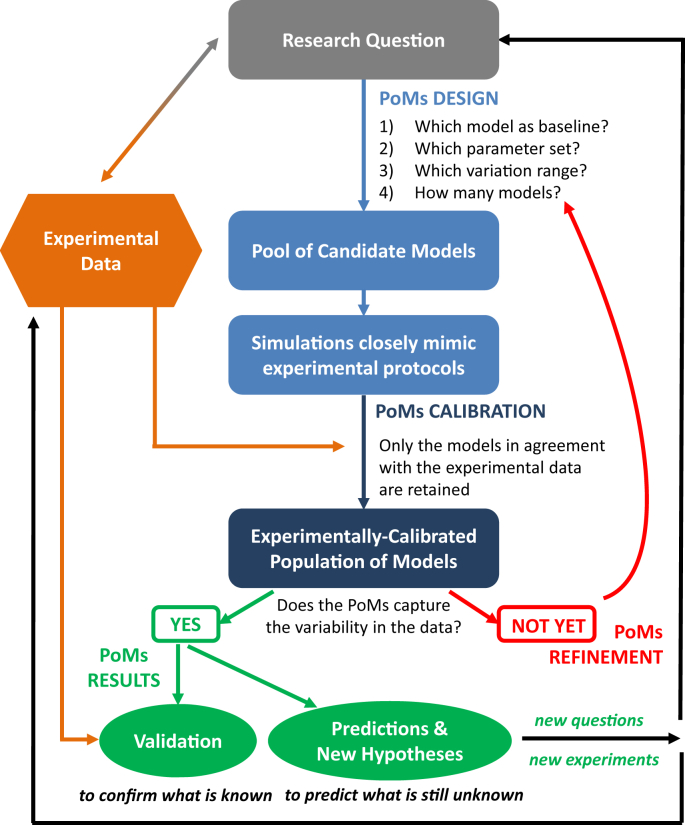
Flowchart illustrating the process behind constructing an experimentally-calibrated population of models (abbreviated as PoMs).

Fig. 1 illustrates the process of developing and analysing an experimentally-calibrated population of models, described in more detail in the following sections.

### 2.1 The research question and the baseline model of cellular electrophysiology

The research question (and corresponding hypotheses) will inform both the choice of experimental data and the modelling process. These will be the two corner stones for the construction of the experimentally-calibrated population of models. A common assumption is that inter-individual variability affects electrophysiology at the level of ionic current properties (such as the ionic current conductances, time constants of channels opening/closing, and other parameters characterising the currents), and not at the level of ion channel structure (which is represented in the models through equations describing each modelled channel's transitions between gating states) (Britton et al., 2013, Groenendaal et al., 2015, Sarkar et al., 2012). Therefore, at the initial stage of modelling, one selects an appropriate cardiac cell model whose model equations are used as a ‘scaffold’, whilst the baseline model parameters are varied to represent variability in ionic current properties.

Aside from the research question, additional factors that may play a critical role in selecting the baseline model to use as the scaffold are model complexity and unique model characteristics, particularly if multiple models of a particular cell type exist. For instance, there are six published biophysically-detailed models of human atrial electrophysiology (Colman et al., 2013, Courtemanche et al., 1998, Grandi et al., 2011, Koivumäki et al., 2011, Maleckar et al., 2009, Nygren et al., 1998). The Colman et al., Courtemanche et al. and Grandi et al. models produce a spike-and-dome AP; however, the latter model includes a formulation for chloride current that is missing in the former. In comparison, the models of Nygren et al., Maleckar et al. and Koivumäki et al. generate more triangular APs. At the same time, the Maleckar et al. model is the only one able to incorporate the effects of vagal stimulation on the AP due to the inclusion of acetylcholine-activated potassium current, while the Koivumäki et al. model contains a much more detailed description of the intracellular calcium transient compared to the remaining models. The assumptions made in a particular study, together with key features of experimental data to be modelled, will determine the relative importance of the model-specific properties for answering the research question (for instance, do experimental APs display a spike-and-dome morphology? How finely-detailed does the calcium subsystem need to be?).

### 2.2 Generating the population of candidate models

Populations of models extend cardiac modelling beyond the use of a single model that represents the average or typical behaviour of that cell type and species, to an ensemble of models with different values of underlying parameters and differing behaviours of model outputs. The choice of parameters to vary is therefore a significant part of designing a population of models.

A parameter should only be varied if an argument, based on the aims of the study, can be made as to why it should be varied. While many parameters within a model of a biological system will exhibit variability in experiments, different biological parameters vary through different mechanisms. For example, both ionic current conductances and kinetics can vary between individuals; however, conductances vary primarily due to variation in the number of ion channels in the cell membrane, while kinetics vary primarily due to changes in channel structure. Genetic mutations that alter channel kinetics and functionality are generally considered pathological, and differentiate individuals from the range of the population that is considered as healthy. Therefore, a population created by varying channel kinetics would have a different biological interpretation from a population created by varying conductances alone.

After determining the parameters that will be varied for the population of candidate models, the next choice is to determine the range over which to sample parameter values. Ranges for parameter sampling should be chosen according to the hypothesis of a particular study and the assumptions made about what the resultant population will represent. Wider parameter ranges allow for more extreme parameter values and therefore more extreme model behaviours. For ionic current conductances, expanding the range towards large values could represent increased expression or activity of that current, as might occur in compensation for block of another current (Marder and Taylor, 2011), or due to beta-adrenergic stimulation (Heijman et al., 2013). Expanding the range towards zero could represent decreased expression of an ion channel, but very low values could also represent mutations that cause loss-of-function of a channel (Rosati and McKinnon, 2004), or diseases that impair channel function (Borlak and Thum, 2003, Farid et al., 2011).

A common step in modelling studies to date is to assume that ionic current conductances vary significantly between individuals and constitute the major determinant behind physiological variability (Britton et al., 2013, Groenendaal et al., 2015, Sarkar et al., 2012). For modelling of non-diseased, healthy cardiomyocytes, the results of previous modelling studies (Corrias et al., 2011, Romero et al., 2009) suggest that a variation of approximately ±30% in conductance values (from the value of the parameter in the baseline model) might be a moderate estimate on the level of variability, while ±50% would allow substantial variability. For studies that are interested in extreme behaviours, particularly including conditions where one or more types of ion channel may be almost completely inhibited or not present in the cell, ±100% variation in ionic current conductances could be used (Britton et al., 2013).

Once the range of parameter variation has been established, the next step in the experimentally-calibrated population-of-models method is to generate a large number of parameter sets for the pool of candidate models, sampled from a high-dimensional parameter space. Sampling every possible combination of parameter values is computationally infeasible given the complexity of most cardiac cell models. Most studies of experimentally-calibrated populations of models in cardiac electrophysiology (Britton et al., 2014, Britton et al., 2013, Muszkiewicz et al., 2014, Passini et al., 2015, Zhou et al., 2013) used Latin Hypercube sampling (Burrage et al., 2015, McKay et al., 1979), a parameter sampling method that does not scale the computational cost with the number of varied parameters, allowing for an exploration of a complex parameter space.

Different techniques could be used to construct an experimentally-calibrated population of models, and an important question is how effective the present methodology is in yielding an ensemble of models that captures the desired features of experimental data. An argument can be made that Latin Hypercube sampling is a crude mechanism for exploring a high-dimensional parameter space and other statistical techniques may be more effective. Alternative approaches such as Approximate Bayesian Computation (ABC) could indeed be considered to perform Bayesian statistical inference for models that do not possess a computationally tractable likelihood function (Beaumont et al., 2002). ABC algorithms search the parameter space until they find a certain number of parameters generating simulated data that are close to the observed experimental data with respect to some summary statistics. In this manner, the ABC identifies a compact region in the parameter space that produces simulated data from a statistical model that is as close as possible to the observed data. Conversely, experimentally-calibrated populations of models can cover a wide region of the parameter space with all models treated equally likely. Whereas ABC can be considered statistically stronger, it may be less effective in identifying outliers than the experimentally-calibrated population-of-models approach. For specific research questions, capturing outliers is crucial, especially when we aim to identify unlikely abnormalities in cardiac function such as cardiac arrhythmias.

### 2.3 Simulation and calibration of the candidate population of models

The population of candidate models generated in the previous step is now simulated and calibrated, to select the models whose simulated electrophysiological properties are in range with the same properties in experimental data. This step yields the experimentally-calibrated population of models. It is crucial that the simulations mimic the experimental conditions and protocols as closely as possible, considering important aspects such as ionic concentrations in the bath, stimulation amplitude and stimulation frequency as well as any drugs used in the experiments.

**Fig. 2:**
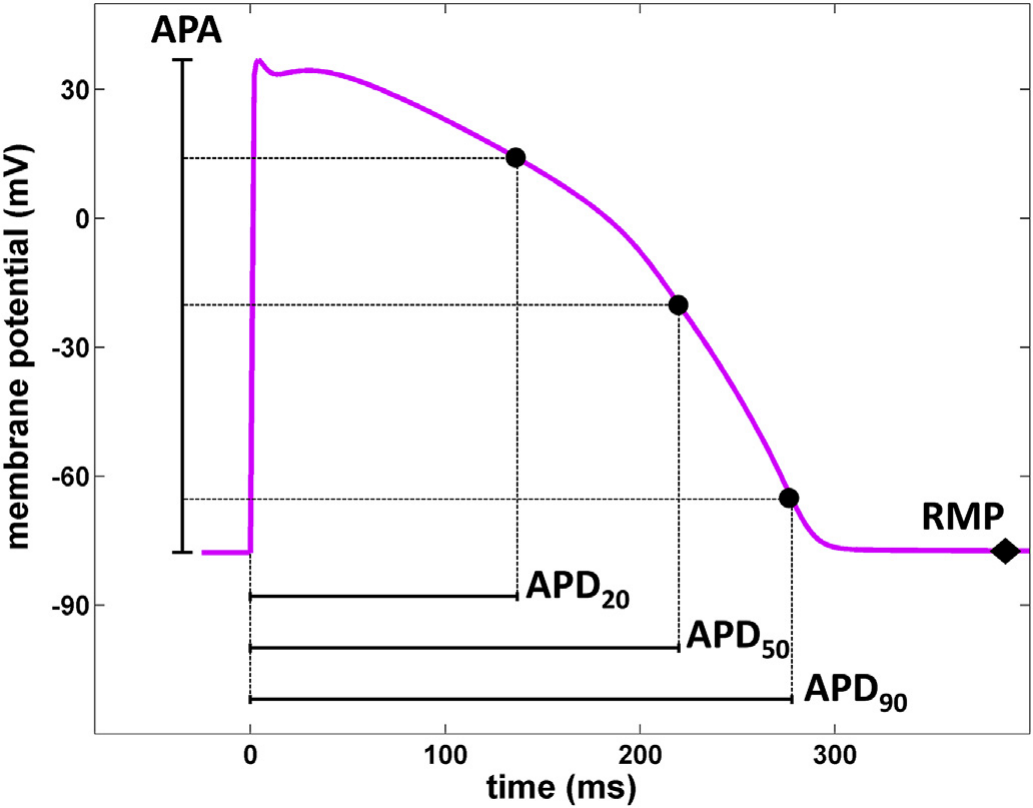
Action potential (AP) trace with some of the typical AP properties utilized in the calibration process. Acronyms: APDxx – action potential duration at xx% repolarization, RMP – resting membrane potential, APA – action potential amplitude.

Calibration criteria naturally differ from study to study, depending on the experimental data available. In some studies, experimental data characterising only the AP properties of cardiomyocytes are provided, while other studies use additional information on the intracellular calcium transient (Passini et al., 2015), and ionic current densities obtained from voltage clamp recordings. Fig. 2 illustrates some of the AP properties that may be used in the calibration process. Table 1 provides a summary of the AP properties and ranges used in different studies, emphasizing consistent variability in the AP across multiple species and sample/tissue types.

In the experimentally-calibrated population-of-models studies to date (Britton et al., 2014, Britton et al., 2013, Muszkiewicz et al., 2014, Passini et al., 2015, Sánchez et al., 2014, Zhou et al., 2013), the experimentally-permitted range of values for model outputs are determined by calculating the minimum and maximum values obtained from experimental measurements. Extreme outliers are often discarded to exclude potential abnormalities due to damaged preparation or dislodged microelectrode impalement. For smaller datasets, this must be done using intuition and experience, as with few data points it is difficult to judge the boundary at which reasonable experimental variation ends. Larger datasets would offer a possibility of refining the way experimentally-permitted calibration criteria are determined. For example, (Prinz et al., 2004) used limits of 2 standard deviations from the mean to set ranges on their calibration criteria for constructing populations of neuronal models. Another approach for refining the calibration step may involve the simultaneous use of different calibration criteria derived from multiple experimental sources (e.g. combining AP-level information with measurements of calcium transient or individual ionic currents, as our most recent work is now exploring (Passini et al., 2015)). Hard limits on model acceptance could be replaced as well by more flexible bounds or likelihood estimators. This would allow analysing the impact of data scarcity, or the matching of specific biomarker distributions, on the predictions generated from different calibrated populations. These aspects of course depend on the availability of sufficiently large experimental datasets, essential for the intended use of the resulting population (to capture the broadest range of possible physiological variability, and in particular outlying phenotypic variants, versus replicating specific or average cell behaviours).

### 2.4 Analysis of the experimentally-calibrated population of models

Analysis of the experimentally-calibrated population presents a number of challenges that differ from those posed by analysis of a single model. Under this methodology, there is no favoured parameter set; instead, all parameter sets within the experimentally-calibrated population are equally valid as all produce output that is consistent with the range seen in experimental data. Additionally, the underlying distribution of varied parameters in the population is determined by the calibration criteria applied to the model outputs. Experimental data for the distributions or ranges of the underlying parameters are usually unavailable and so the parameter distributions underpinning the experimentally-calibrated population reflect the selectivity of the calibration criteria and experimental data used, rather than direct experimental measurements of the parameter distributions.

**Fig. 3:**
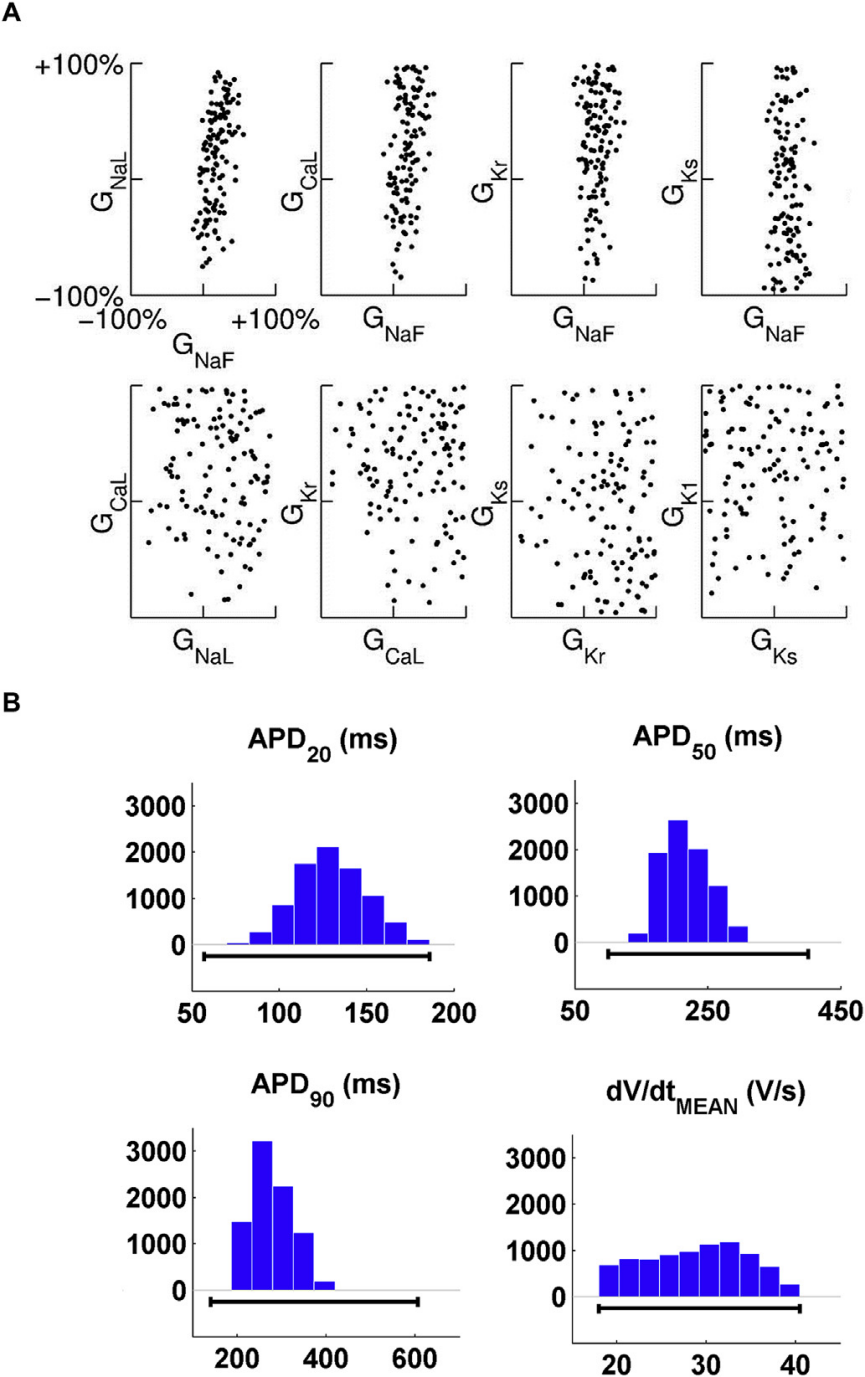
Visualizations helpful for understanding the properties of the experimentally-calibrated population of models include scatter plots and histograms summarising the population's properties without assuming a particular probability distribution for the simulated data. (A) Scatter plots of ionic properties underpinning the experimentally-calibrated population in (Britton et al., 2013), with the scale in all graphs including ±100% variation with respect to the baseline model value. (B) Histograms illustrating the distribution of the AP properties across the experimentally-calibrated population of non-diseased human ventricular myocytes in (Passini et al., 2015). Histograms show the number of models for each of the bins. Black lines indicate the experimental range used to calibrate the population for each AP property.

A first step for understanding the properties of the experimentally-calibrated population is visualisation of the underlying parameters and model outputs. Useful tools are scatter plots (Fig. 3A), to examine correlations between pairs of variables such as ionic conductances; as well as histograms and boxplots, to summarise the distributions of parameters and model outputs without assuming a particular probability distribution for the simulated data (Fig. 3B). Direct visualisation of the high-dimensional parameter and model output spaces is difficult. However, machine learning techniques such as principal component analysis can be used to identify covariance between multiple variables, while other techniques such as clutter-based dimension reordering (Gemmell et al., 2014, LeBlanc et al., 1990, Peng, 2005, Peng et al., 2004, Taylor et al., 2006) are available to represent high-dimensional data in a single image.

It should be noted that the number of models in the experimentally-calibrated population is usually larger than the number of experiments used to calibrate the population, as well as the number of experiments used to validate model predictions. This is because the aim of the virtual population is to capture as much of the experimental range of variability seen across the dataset as possible, and to explore a large number of possible variant models consistent with experiment.

## 3 Novel insights from experimentally-calibrated population-of-models studies to date

Once a population's basic properties have been analysed, the population is often simulated in conditions beyond those that were used to develop it, and the results used as predictions of the population, or to generate new hypotheses. A traditional single model's prediction would either be treated as a qualitative one (e.g. whether the AP duration (APD) increases, decreases, or stays the same following the application of a drug), or tested to see whether it was close to the mean value from experiment (by comparing to the standard deviation of the experimental data). In contrast, an experimentally-calibrated population of models can be used to generate quantitative predictions of the range of effects expected in a given intervention that are directly comparable to experimental data. A prediction on the range of effects can be more informative than a prediction of the average behaviour in cases where outlier responses are important (for instance in safety pharmacology).

In some studies, the focus is not on understanding the mechanisms behind purely quantitative changes such as APD prolongation due to drug application. Instead, different cell groups may show qualitatively different responses to the same intervention. For example, the same drug may trigger abnormal repolarizations in cardiomyocytes from one individual, while cells from another subject will repolarize normally. In this case, the aim of the analysis is to identify sub-populations of models within the experimentally-calibrated population that display the behaviour of particular interest, and determine whether any combinations of parameters can be used to distinguish this sub-population from the remaining models. Differences identified in this manner can then be used to inform new hypotheses regarding the plausible mechanisms causing a particular behaviour to occur. Standard methods for single model analysis can be used on exemplar models within the sub-population to provide evidence for whether a proposed mechanism is responsible for the observed behaviour. The methods that could be used here include detailed analysis of state variables, to establish cause-and-effect in the model; as well as bifurcation analysis, to analyse the different dynamical states a particular model can occupy.

### 3.1 Variability under physiological conditions

To capture as much variability as possible, populations of candidate models can be calibrated with data from a wide range of sources, for instance using the existing experimental literature to define a range of values for the properties characterising the AP. This is well-illustrated with the experimentally-calibrated population of rabbit ventricular myocyte models constructed in (Gemmell et al., 2014), where a literature search identified 13 different sources of AP data and was sufficient to establish a physiological range for APD90, a commonly-reported AP property (Table 1). Less commonly-reported properties, such as APD50, required a different approach: in this case, the literature was used to calculate mean APD50, but its physiological range of values was estimated on the assumption that its percentage variation from the mean was identical to that for APD90.

In this way, the population of rabbit ventricular models was calibrated with experimental data from multiple sources, and encompassed variation observed across a variety of experimental samples. Conclusions drawn from such a population are not specific to a particular experiment or a narrow group of cells. Instead, such a population inherently incorporates determinants and modulators of variability that are both intrinsic and extrinsic to the experimental preparations (for instance, ionic current properties versus differing ionic concentrations in the bath due to modifications in experimental protocol). Analysis of the experimentally-calibrated population by Gemmel et al. revealed interesting correlations between the underpinning ionic current properties. For example, the experimentally-calibrated population based on the (Shannon et al., 2004) model of rabbit ventricular electrophysiology tended to require reductions in the conductances of I_CaL_ and I_K1_ (with respect to the baseline model), and while the population overall covered an even range of values for conductances of I_NaK_ and I_Kr_, these two parameters were correlated, with an increase in I_NaK_ corresponding to an increase in I_Kr_.

At the other end of the spectrum, the experimentally-calibrated population-of-models approach has also been used to construct models mimicking the behaviour of cells extracted from a narrow patient group. This is illustrated by (Muszkiewicz et al., 2014), who used a rich experimental dataset consisting of AP recordings of human atrial myocytes obtained from right atrial appendages of elderly male patients, all of whom were in sinus rhythm and undergoing a common clinical procedure in the same geographical location (Liu et al., 2013). The AP properties elicited from these cells were quite distinct from those generated by the baseline models of human atrial electrophysiology published in the literature, with APD90 spanning 63–143 ms (Table 1), compared to the conventional model values between 197 and 330 ms (Colman et al., 2013, Wilhelms et al., 2012).

Muszkiewicz et al. constructed a population of models that mimicked the range of variability exhibited by the AP properties pertaining to this narrow cell group, and investigated the relative impact of the sources of variability intrinsic and extrinsic to the cell on AP generation (Muszkiewicz et al., 2014). Specifically, variation in ionic current conductances constituted an intrinsic source of variability, while stimulation amplitude and ionic concentrations comprised extrinsic variability. The authors showed that variability in the early repolarization stage in the models was highly influenced by the stimulus strength, whereas that in resting membrane potential was critically dependent on ionic concentrations. Simultaneously, intrinsic variability represented by the variation in ionic current conductances was key to capturing the range of APD90 values observed in experiments. These findings suggest that, to construct a population of models mimicking a narrow cell group, one may need to consider variability in extrinsic factors in addition to variability in ionic current properties. In turn, this may have important implications for understanding the propensity of different patient subgroups to disease conditions.

### 3.2 Variability under pathological conditions

**Fig. 4:**
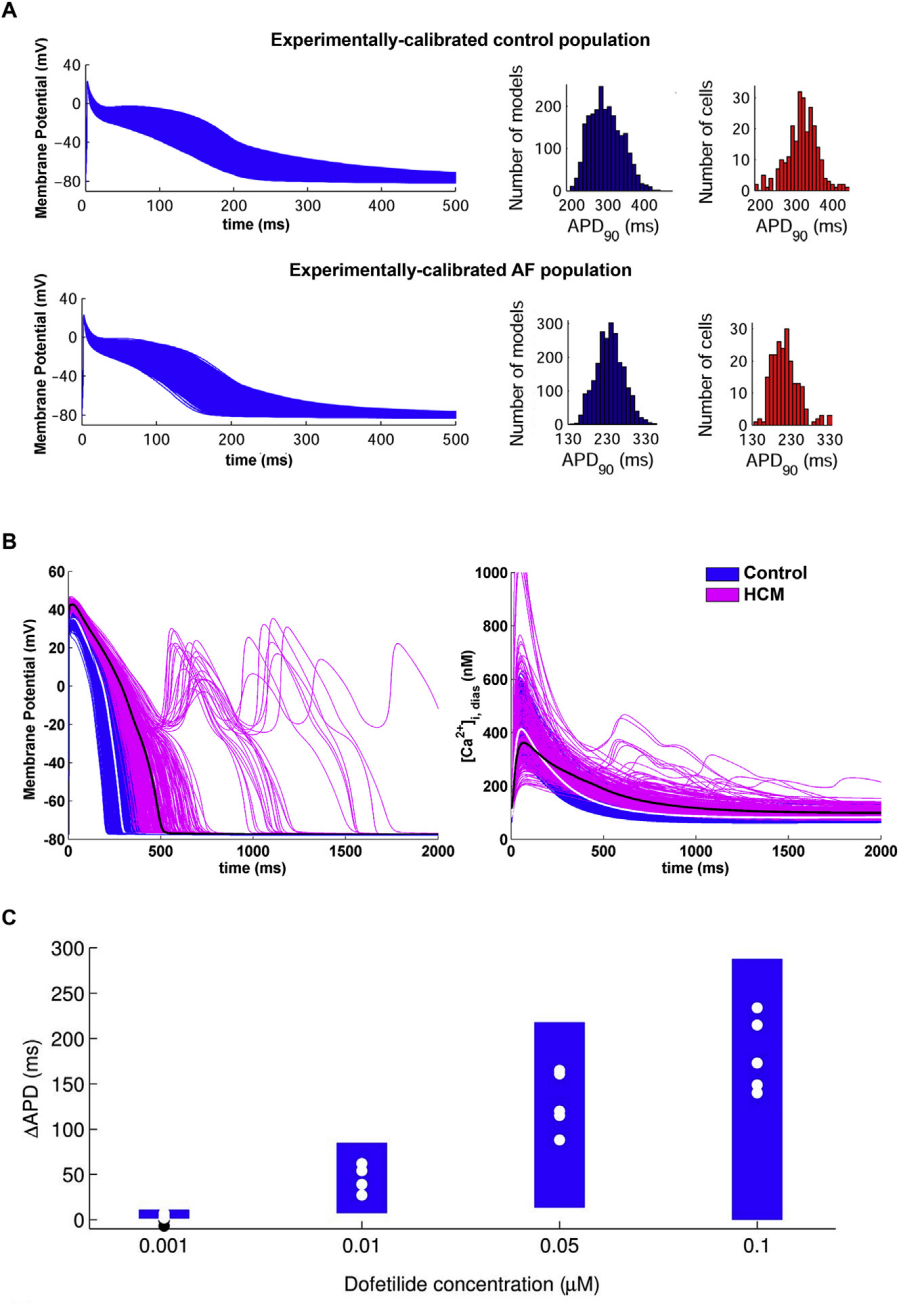
Experimentally-calibrated populations of models applied to investigations of physiological variability in (A) atrial fibrillation, (B) hypertrophic cardiomyopathy, and (C) under drug action. (A) AP traces of experimentally-calibrated populations of models mimicking control (top) and diseased cells (bottom), including histograms of APD90 in simulated (blue) and experimental data (red), modified from (Sánchez et al., 2014)). (B) AP traces (left) and intracellular calcium transients (right) in experimentally-calibrated populations of models mimicking control (blue) and diseased cells (pink); white and black traces illustrate the output of the baseline model in the absence and presence of HCM-induced electrical remodelling. Reproduced from (Passini et al., 2015)). (C) Ranges of APD prolongation (ΔAPD) caused by four concentrations of potassium current blocker dofetilide in the models comprising the experimentally-calibrated population in (Britton et al., 2013); dots indicate values of ΔAPD obtained independently in five experiments using rabbit Purkinje fibre preparations. Reproduced from (Britton et al., 2013).

Experimentally-calibrated populations of models can be useful to better understand the ionic mechanisms underlying particular cardiovascular diseases. As an example, atrial fibrillation (AF) is the most commonly diagnosed cardiac arrhythmia, but mechanisms of its generation and maintenance are still a matter of debate. Furthermore, phenotypic variability in human atrial electrophysiology in AF is large, overlapping in many cases with that of healthy subjects in sinus rhythm used as control (Table 1). In a recent study, populations of models were calibrated with experimental datasets from over 450 cell samples extracted from patients in sinus rhythm and AF, in order to investigate the ionic mechanisms underlying inter-subject variability in human atrial AP properties between AF and control cells (Sánchez et al., 2014). Despite intrinsic differences between the populations representing control and AF scenarios (Fig. 4A), both have shown similar mechanisms underlying variability in different stages of the AP: I_CaL_, I_to_ and I_Kur_ were found to be key in modulating inter-subject differences in early repolarization, whereas I_K1_ and I_NaK_ determine cell-specific values of APD90. These findings may help in understanding inter-subject differences in human atrial dynamics and the response to anti-AF pharmacological therapies.

**Fig. 5:**
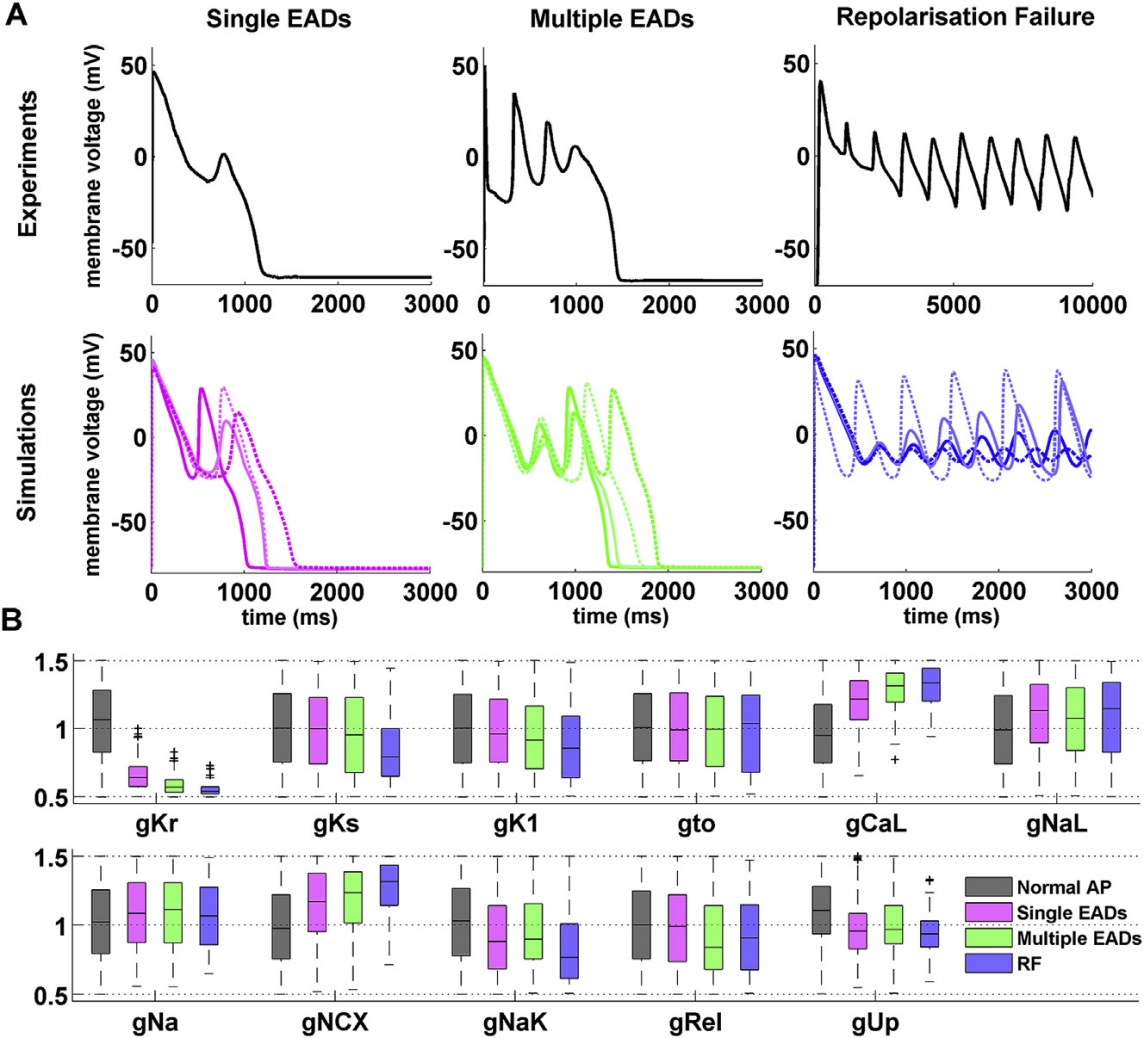
Experimentally-calibrated population of human HCM models consists of three sub-populations, including models with a single EAD, multiple EADs, and repolarization failure. (A) Representative experimental (top) and simulated (bottom) HCM action potential traces, showing the three types of repolarization abnormalities. (B) Normalized distributions of ionic properties for the 11 ionic current conductances varied within the population, for models displaying normal AP (n = 8366), single EADs (n = 480), multiple EADs (n = 201), and repolarization failure (RF, n = 71). In each box, the central line represents the median, the box limits correspond to the 25th and 75th percentiles, the whiskers extend to the most extreme data points not considered as outliers, while the outliers are depicted individually as crosses. Reproduced from (Passini et al., 2015).

The experimentally-calibrated population-of-models approach has also been used to investigate hypertrophic cardiomyopathy (HCM) (Passini et al., 2015), a cardiac genetic disease characterised by an increased arrhythmic risk and still lacking a specific pharmacological treatment. Based on human experimental data ((Coppini et al., 2013); Table 1), two populations of models were constructed representing both control and HCM cardiomyocytes (Fig. 4B). The electrical remodelling induced by HCM was subsequently investigated by analysing the different ionic currents and Ca^2+^ subsystem changes, and the electrophysiological phenotype of the disease successfully reproduced in the population of human ventricular HCM cells. Consistent with experimental findings, the study identified three distinct sub-populations within the experimentally-calibrated population of HCM models, including models displaying a single or multiple EADs as well as models failing to repolarize (Fig. 5A). Pro-arrhythmic mechanisms within these sub-populations were subsequently investigated by analysing their underpinning ionic current conductances (Fig. 5B). In this way, common ionic mechanisms contributing to EADs generation were identified, thus suggesting potential therapeutic targets in human HCM. Based on these findings, selective and multi-channel current blocks were tested in simulations, giving new insights about potential anti-arrhythmic drugs for the pharmacological management of the disease, which may be able to suppress repolarisation abnormalities, as well as reverse the HCM phenotype. Overall, these findings illustrate the power of the experimentally-calibrated population-of-models approach to predict and explain disease phenotype, provide the means for a quantitative comparison between experimental and simulation data, and investigate potential disease therapies.

Zhou et al. (Zhou et al., 2013, Zhou et al., 2015) also applied the experimentally-calibrated population-of-models approach to study mechanisms underpinning cardiac alternans, which are stable beat-to-beat fluctuations between subsequent APs elicited from the same cell. While cardiac alternans have an unequivocal connection to arrhythmia onset (Rosenbaum et al., 1994), most investigations of alternans to date were based on animal experiments, providing the need for a human-focused study to avoid inter-species differences in electrophysiology. To investigate the mechanisms underlying alternans generation in human cardiomyocytes *in vivo*, a population of human ventricular cell models was generated to include the effect of natural variability in ionic current conductances. Activation recovery intervals (ARIs) were calculated from *in vivo* electrograms obtained using epicardial socks (Nash et al., 2006, Taggart et al., 2014) from 41 patients undergoing coronary cardiac surgery. The ARIs were used as *in vivo* surrogate of APD90 during the calibration process (Table 1) to select the ventricular cell models exhibiting physiological APD ranges at six different pacing rates (Zhou et al., 2013, Zhou et al., 2015). The *in silico* population of human ventricular cell models successfully reproduced the frequency dependence of repolarization alternans as observed in the *in vivo* recordings. Further analysis of the human alternans models revealed the key role of fluctuations in the sarcoplasmic reticulum calcium content in the initiation of alternans, regardless of differences in ionic currents. The sodium calcium exchanger functioned as the main translator between calcium fluctuation and APD alternans, and its modulation was shown to be an effective anti-arrhythmic strategy for the management of cardiac alternans.

### 3.3 Variability in response to drug action

Ensembles of models were used to investigate the role of variability in modulation of repolarization reserve and response to ionic channel block (Cummins et al., 2014, Devenyi and Sobie, 2015, Sarkar et al., 2012, Sobie and Sarkar, 2011, Sobie, 2009). Britton et al. (2013) proposed the methodology to construct and calibrate a population of models using experimental data, and then evaluate predictions of this experimentally-calibrated population following drug block. Specifically, (Britton et al., 2013) built a population of models mimicking variability in the AP properties of isolated rabbit Purkinje fibres under control conditions (Table 1), and used the resultant population to investigate the effect of four concentrations of dofetilide, a selective I_Kr_ blocker, on rabbit ventricular AP. The ranges of APD prolongation predicted by the population were found to be in agreement with blinded experimental results that were not used to construct the population (Fig. 4C). This demonstrates the ability of the population-of-models methodology to make quantitative predictions that can be compared directly to experimental results, and to connect drug effects to specific ionic mechanisms. Additionally, this achievement illustrates the inherent potential of the methodology to meet the industry needs for safety pharmacology testing, as for the replacement, refinement and reduction (3Rs) of existing animal assays in this industrial sector.

In addition to APD prolongation due to potassium channel blocker, the experimentally-calibrated population-of-models approach has also been used to study pro-arrhythmic effects of drugs (Britton et al., 2014). In some circumstances, drug application can lead to the development of abnormalities in repolarisation at the cellular level, including EADs (Lu et al., 2002, Savelieva and Camm, 2008). Therefore, (Britton et al., 2014) used an experimentally-calibrated population to investigate the relative importance of sarcolemmal currents in determining the susceptibility of human ventricular cardiomyocytes to drug-induced repolarisation abnormalities for specific and multiple ionic current blocks. Every model within the experimentally-calibrated population produced action potentials consistent with normal pacing under control conditions; however, the response to inhibition of the repolarising currents I_Kr_, I_Ks_ and I_K1_, along with I_CaL_, widely accepted as a key mechanism of EAD generation (January and Riddle, 1989), was highly heterogeneous across the population. The conductances of I_CaL_ and I_NaK_ were identified as crucial for determining whether a given model developed drug-induced repolarisation abnormalities across a wide range of repolarising current block levels. Specifically, moderate to high I_CaL_ and very low values of I_NaK_ were present in models that were identified as highly susceptible to repolarization abnormalities or repolarization failure.

## 4 Discussion

In this paper, we have analysed the scientific considerations behind the construction of experimentally-calibrated populations of models as a novel conceptual framework for investigating the impact of variability on cardiac electrophysiology. The utility of such an approach is further exemplified through a detailed review of different studies employing this methodology, to investigate variability in cardiac function in both healthy and pathological conditions and under drug action. The methodology is also compared to alternative approaches for studying variability, including the sensitivity analysis and cell-specific modelling methods.

The experimentally-calibrated population-of-models approach merges models, simulations and experiments into a tightly interconnected system (Carusi et al., 2012) that can be used to generate new insights and hypotheses concerning variability observed in a particular experimental dataset, as illustrated in Fig. 1. In turn, these insights can be fine-tuned and tested via additional experiments and refined models, deepening our understanding of variability and its impact on cardiac electrophysiology. In this way, experimentalists and modellers work closely with each other, resulting in a modelling approach that is more responsive to experimentalists' concerns, and more likely to generate hypotheses that can be experimentally tested (Carusi, 2014, Quinn and Kohl, 2013, Quinn and Kohl, 2011). Therefore, in the population-of-models approach, models are regarded as tools to probe variability in cardiac electrophysiology (Boon and Knuuttila, 2009, Carusi et al., 2012).

### 4.1 Importance of variability in biology and medicine

Understanding inter-subject and intra-subject variability in physiological and pathological function is one of the biggest challenges in biology and medicine.

Sex and age differences have been identified as important determinants of inter-subject variability (Abi-Gerges et al., 2006, Abi-Gerges et al., 2004, Gaborit et al., 2010, Ganjehei et al., 2011, Herraiz-Martínez et al., 2015, Sims et al., 2008, Villareal et al., 2001). For instance, (Abi-Gerges et al., 2006, Abi-Gerges et al., 2004) discussed differences in cardiac electrophysiology and inducibility of ventricular arrhythmias between females and males, while (Rosano et al., 1996) showed that susceptibility to paroxysmal supraventricular tachycardia in women varies with changes in hormonal levels during menstrual cycle. Additionally, (Sims et al., 2008) demonstrated that sex, age, and regional differences in expression of I_CaL_ ionic current are determinants of arrhythmia phenotype in rabbit with drug-induced long QT syndrome, while (Herraiz-Martínez et al., 2015) demonstrated that the amplitude of intracellular calcium transient in atrial cardiomyocytes decreased with age.

Intra-subject variability in electrophysiology manifests itself as spatial heterogeneities (including differences in repolarization from different regions of the heart, see for instance (Bueno-Orovio et al., 2012)). This variability within the same individual can also be modulated temporarily by, for example, heterogeneous nervous innervation or circadian rhythms. In mice, circadian rhythms were found to alter protein expression of an important I_to_ subunit by approximately ±40% from the midpoint over a 24 h cycle (Jeyaraj et al., 2012). Beat-to-beat variability in the AP is another manifestation of temporal variability, and processes such as alternans and the stochastic nature of ion channel dynamics have been implicated (Heijman et al., 2013, Pueyo et al., 2011, Walmsley et al., 2015).

Besides, physiological variability in humans is expected to be larger than in animal models, given the controlled homogeneity of certain animal lines used in research juxtaposed with the highly heterogeneous nature of any human population (in terms of lifestyle, age, and genetics). This advocates studies into (i) the sources and modulators of physiological variability in humans, as well as (ii) the implications of this variability in disease and treatment. A significant portion of further studies using the population-of-models approach can be aimed at characterising variability in specific subgroups of human patients based on age and sex (as in (Yang and Clancy, 2012)), as well as the determinants of spatio-temporal intra-subject variability.

### 4.2 Ionic determinants of variability in cardiac electrophysiology

A central assumption in modelling studies of physiological variability in cardiac electrophysiology is that the properties of ionic currents, such as their conductances, are the main determinants of variability. Multiple processes affecting transcription, translation, degradation and circadian rhythms could impact ionic current conductances and contribute to physiological variability in ionic currents and the AP. Documented differences exist between transcription rate and mRNA level, mRNA level and protein level, and translation rate and protein level (see Fig. 1 in Shu and Hong-Hui, 2004). For instance, mRNA expression of genes that code for ion channel proteins is influenced by post-transcriptional regulation and buffering through processes that translate mRNA to functional ion channels (Nattel et al., 2010). Further, there is evidence that mRNA expression can vary by large amounts, from almost complete downregulation up to increases of at least four-fold compared to baseline (Borlak and Thum, 2003) in the most extreme cases of remodelling such as heart failure.

It is crucial to emphasise that the range of variation in ionic current properties cannot be determined in experiments, as (i) when elicited under standard laboratory protocols, the AP traces alone do not carry enough information to pinpoint all ionic current properties, and (ii) ionic currents can only be measured in voltage-clamp experiments on isolated cardiomyocytes. The inherent limitation of voltage-clamp protocol is its adverse impact on ion channel density, known to be affected by the cell isolation procedures (Yue et al., 1996). Furthermore, if measurements of all ionic current conductances in a specific preparation and at a particular point in time could be possible, ionic conductances are subject to continuous variation caused by extrinsic factors (Sánchez et al., 2014) that include long-term drug effects (Xiao et al., 2008) and circadian rhythms (Jeyaraj et al., 2012). The experimentally-calibrated population-of-models methodology allows us to identify and probe key determinants of physiological variability at the ionic level, and suggest a plausible range of their variation. In fact, a study by (Weiss et al., 2012) showed the correspondence between gene expression data and a population of computer models of the mouse ventricular action potential, supporting the hypothesis that biological systems are generally ‘sloppy’ (Gutenkunst et al., 2007), i.e. tolerant to significant variations of many parameter combinations.

At the level of single cells, physiological variability may manifest itself not only via differences in ionic current conductances and kinetics, but through differences in other variables impacting ionic current balance. For instance, inter-cellular variability in cardiomyocyte volume and membrane capacitance has been linked to patients' age (Polak and Fijorek, 2012). Variability in extracellular and intracellular ionic concentrations has been shown to have an effect on the AP-level properties such as the resting membrane potential and APD90 (Muszkiewicz et al., 2014, Passini et al., 2014, Vincenti et al., 2014).

### 4.3 Comparison of the experimentally-calibrated populations of models with other approaches for studying variability

**Fig. 6:**
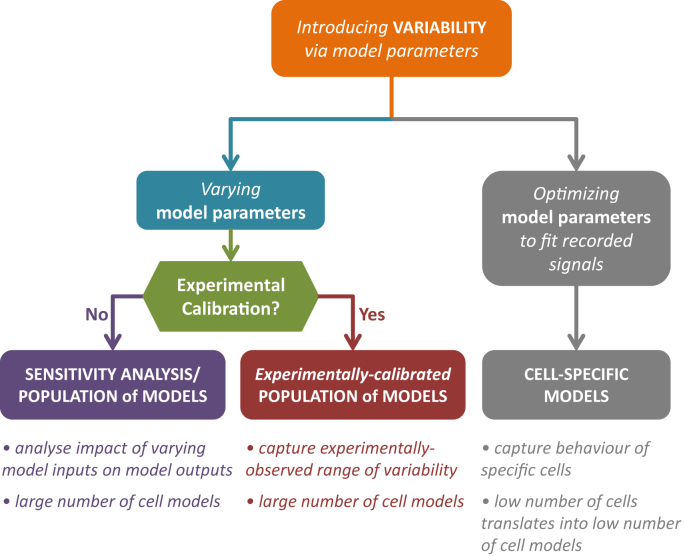
Schematic illustrating the different approaches for studying physiological variability.

In addition to the experimentally-calibrated population-of-models approach, three other frameworks for computational studies of cell-to-cell physiological variability in cardiac electrophysiology have been devised: the sensitivity-analysis method, the population or ensemble-of-models framework without experimental calibration, and cell-specific modelling approaches (Fig. 6). Sensitivity-analysis-based methods explore the effect of variation in model parameters on model outputs such as the action potential or intracellular calcium transient. The parameters of interest are varied around set values – usually the baseline model values – one at a time. Univariate sensitivity analysis was used by Romero et al. to investigate variability by varying one parameter at a time by ±15% and ±30% from its baseline value, and to explore the effect this had on pre-clinical biomarkers of arrhythmic risk, including the APD, in a human ventricular single-cell model (Romero et al., 2009).

In the absence of experimental calibration, the population-of-models approach can be thought of as an extension of the sensitivity-analysis framework, in that it permits multiple parameters to be varied simultaneously, and in the context of cardiac electrophysiology, it was first used in a series of papers by (Sobie and Sarkar, 2011, Sobie, 2009). There, multiple parameters were varied simultaneously in models of ventricular cells, with parameter values sampled from a log-normal distribution centred on their baseline values. Then, the authors used regression analysis to create simplified models relating changes in parameters describing ionic currents to changes in cellular properties. Using similar methods, Walmsley et al. investigated mRNA expression levels on the cellular electrophysiological remodelling in failing human hearts (Walmsley et al., 2013, Yang and Clancy, 2012) probed sex-based differences in human susceptibility to cardiac ventricular tachyarrhythmias; and Cummins et al. compared rate-dependent properties of 13 models of ventricular cells in the context of drug action (Cummins et al., 2014). Most recently, the methodology was used by (Devenyi and Sobie, 2015), who studied regulation of calcium transient in rat cardiomyocytes via iterations with experiments.

Both the sensitivity-analysis approach and the population-of-models framework without experimental calibration are very useful as tools to probe the relationships between ionic current properties and cellular properties such as the AP. However, unlike the experimentally-calibrated population-of-models methodology, they do not incorporate a calibration step permitting one to constrain models with experimental data. Therefore, they cannot generate models representative of datasets extracted from various patient or subject groups. This is important, as experimentally-calibrated populations-of-models provide a framework to quantitatively (rather than qualitatively) compare simulation and experimental predictions (Fig. 5). Additionally, narrow ranges of parameter variation combined with the absence of calibration criteria mean that the sensitivity-analysis and ensemble-of-models frameworks cannot be used to generate models whose predictions are far from the baseline model value, while at the same time mimicking the experimental recordings.

In cell-specific modelling approaches, the aim is to generate biophysically-detailed models of specific cells used in experiments. Syed et al. were the first to illustrate this approach by using a genetic algorithm (Syed et al., 2005) to fit a biophysically-detailed model of human atrial action potential to the AP trace generated by different cell models, as well as by experimental data. Separately, Davies et al. constructed 19 models of 19 dog ventricular mid-myocardium myocytes. The models were fitted to the action potential recordings in the early and late stages of cellular repolarization under control conditions; a subsequent comparison of model predictions to experimental observations of drug action revealed that models had a prediction accuracy of over 80% (Davies et al., 2012). Therefore, the approach proved useful; however, the fact that the experimental AP traces used to constrain the models were collected with standard laboratory protocols implies that the uniqueness of parameter values in the resultant cell models cannot be guaranteed. Improvements to the methodology were put forward by Kaur et al. (2014) and Groenendaal et al. (2015). Kaur et al. demonstrated that incorporating membrane resistance alongside action potential measurements led to faster convergence in the estimation of cell specific model parameters. Groenendaal et al. developed a series of complex electrophysiology protocols involving stochastic pacing of the cells in experiments, coupled with the use of automated parameter optimization techniques, to construct 4 specific models of 4 isolated guinea pig ventricular myocytes. Cell-specific modelling could be a powerful approach to address specific research questions, also in line with a tightly interconnected model-simulation-experiment system. However, the complexity of the protocols involved limits the number of cells that can be investigated and modelled, and therefore it does not provide the wide coverage of the parameter space of the experimentally-calibrated populations of models.

### 4.4 Experimentally-calibrated population-of-models approach beyond the heart

Experimentally-calibrated populations of models have also been used in areas outside of cardiac electrophysiology. The approach was originally pioneered by Marder et al. (see, for instance, (Marder and Taylor, 2011, Prinz et al., 2004)) in neuroscience. In particular, Prinz et al. constructed an experimentally-calibrated population containing over 20 million models of a three-neuron network, with varied synapse strengths and ionic current conductances (Prinz et al., 2004). The authors subsequently used this population to demonstrate that similar patterns of neuronal network activity can arise from widely disparate sets of underlying mechanisms. In a different study, Kispersky et al. investigated the impact of the sodium conductance values on the frequency–current relationships in neurons via an experimentally-calibrated population of neuronal models. They found that increased sodium current conductance led to higher neuronal firing rates for low input currents; however, for large input currents an increase in sodium conductance lowered the firing rate due to altered potassium current availability (Kispersky et al., 2012). The experimentally-calibrated population-of-models approach has also been used in the field of computational biomechanics by Sierra et al. (2015), who probed mechanical properties of skeletal muscle. These studies demonstrate the universal applicability of the experimentally-calibrated population-of-models approach for investigating variability.

## 5 Conclusions and future directions

Cardiac electrophysiology constitutes one of many scientific disciplines that have historically been focused on understanding the average properties of the system under investigation. However, increasing evidence points towards the crucial role of inter-subject and intra-subject differences in cardiac electrophysiological function, particularly in pathological conditions. Understanding this physiological variability is one of the biggest challenges in biology and medicine, and probing variability is difficult with experimental methods alone. In recent years, different computational modelling frameworks have been devised to study physiological variability in cardiac electrophysiology. One such framework uses experimentally-calibrated populations of models, i.e. ensembles of computational models constrained with experimental data, to study causes and drivers of physiological variability under a variety of conditions, including disease and drug action. To date, this methodology has been used to investigate inter-subject and intra-subject variability largely with experimentally-calibrated populations of models of isolated cardiomyocytes.

Future work will involve extending the methodology to multicellular simulations, taking the studies beyond the single-cell level, as in Sánchez et al. (2013). This is especially important in pathological conditions: while variability is generally reduced when cells are coupled in tissue, disease states are known to cause cell-to-cell uncoupling (for instance, reduction in the levels of connexin 43, a membrane protein crucial for electrical communication between cells, has been linked to arrhythmias (Poelzing and Rosenbaum, 2004, Poelzing et al., 2004)). Therefore, we need to understand under what conditions physiological variability becomes pathophysiologically-relevant in tissue and in the whole heart. This review is part of the special issue on Recent Developments in Biophysics and Molecular Biology of Heart Rhythm.

## Editors' note

Please see also related communications in this issue by Clerx et al. (2016) and Ravagli et al. (2016).

## Figures and Tables

**Table 1:** Variability in physiological properties across species, experiment type, tissue/cell type, investigated electrophysiological properties, measurement frequencies (for AP measurement only). Minimum and maximum values of APD90 at 1 Hz are used as illustration of variability. Acronyms: APDxx – action potential duration at xx% repolarization, RMP – resting membrane potential, APA – action potential amplitude, dV/dtMEAN and dV/dtMAX – mean and maximum slope of the AP upstroke, respectively, V20 – voltage measured at 20% of APD90 time, ARI – activation-recovery interval.

Species	Experiment type	Tissue/cell type and cell number	Properties investigated	Frequencies at which experiments performed (for AP measurements only)	APD90 at 1 Hz (unless otherwise specified)		Reference
Min (ms)	Max (ms)
Human	Whole-cell patch-clamp	Isolated atrial cardiomyocytes (n = 29 cells)	APD20, APD50, APD90, RMP, APA	0.25, 0.5, 1, 2, 3 Hz	63.4	131.6	(Liu et al., 2013, Muszkiewicz et al., 2014)
Human	Micro-electrode recordings	Atrial trabeculae from patients in sinus rhythm (n = 254 preparations from 214 patients)	APD20, APD50, APD90, RMP, APA, V_20_, dV/dt_MAX_	1 Hz	193	467	(Sánchez et al., 2014)
Human	Micro-electrode recordings	Atrial trabeculae from patients with chronic atrial fibrillation (n = 215 preparations from 149 patients)	APD20, APD50, APD90, RMP, APA, V_20_, dV/dt_MAX_	1 Hz	141	349	(Sánchez et al., 2014)
Human	Whole-cell patch-clamp	Isolated ventricular non-diseased cardiomyocytes (n = 25 cells)	APD20, APD50, APD90, RMP, APA, dV/dt_MEAN_	0.2, 0.5, 1 Hz	105	687	(Coppini et al., 2013, Passini et al., 2015)
Human	Whole-cell patch-clamp	Isolated ventricular hypertrophic cardiomyopathy cardiomyocytes (n = 80 cells)	APD20, APD50, APD90, RMP, APA, dV/dt_MEAN_	0.2, 0.5, 1 Hz	238	997	(Coppini et al., 2013, Passini et al., 2015)
Human	Micro-electrode recordings	Human right ventricular non-diseased papillary and trabeculae samples (n = 62 preparations from 38 hearts)	Peak voltage, Time of peak voltage, APD40, APD50, APD90, Triangulation 90–40, RMP	1 Hz	178	442	(Britton et al., 2014)
Human	In vivo epicardial sock	In vivo electrograms (240 sites in n = 41 patients)	ARI (as surrogate of APD90)	1.67, 1.82, 2, 2.22, 2.5, 2.86 Hz	149 (at 1.67 Hz)	391 (at 1.67 Hz)	(Zhou et al., 2013, Zhou et al., 2015)
Rabbit	Micro-electrode recordings	Isolated rabbit Purkinje fibres (n = 12 preparations)	APD90, RMP, Peak voltage, dV/dt_MAX_, Plateau Duration, Peak Dome	0.2, 1, 2 Hz	188	342	(Britton et al., 2013)
Rabbit	Isolated ventricular myocytes, left ventricular tissue preparations, and Langendorff-perfused hearts	13 data sources, as identified in systematic literature review	APD90, APD50	1, 1.667, 2.5 Hz	167	230	(Gemmell et al., 2014)
